# Gutta-Percha Stopping

**Published:** 1883-12

**Authors:** Chas. E. Francis

**Affiliations:** New York


### GUTTA-PERCHA STOPPING.

CHAS. E. FRANCIS, D. D. S., NEW YORK.

[NOTE.—Frequent inquiries concerning the practical value of this material and the method of manipulating it, is the writer's excuse for printing the following communication.]

Among the various preparations for filling carious teeth, gutta-percha stopping holds an exceedingly important place.

Cases are commonly presented where defective teeth can be better preserved if filled with this material, than with any other substance. Owing to its nice adaptability to the dentinal walls, together with its slightly expansive nature, it can be made to seal cavities in which it is packed, with a remarkable degree of thoroughness.

For bucco-cervical cavities of second and third molars, it will stand for years, and prove exceedingly effective in preventing renewed decay.

It is frequently and advantageously used for packing against cervical walls of large buccal or approximal cavities, prior to introducing fillings of oxy-phosphate of zinc or amalgam ; also for repairing large gold fillings with cervical borders slightly undermined.

As a stopping for deciduous teeth, it can be quickly introduced, and in most cases answers admirably; also for impoverished or poorly calcified teeth when attacked by caries, and is peculiarly well adapted in cases of white decay, or where the tooth structure is undergoing rapid decalcification.

As a temporary stopping for early decay in permanent teeth, nothing can surpass, or perhaps equal it, for safety. It holds good until the dentine becomes more dense, and the patient older and better able to tolerate the introduction of compact gold fillings.

In cases where the dental pulp is nearly or quite exposed, protection should be afforded by a covering of oxy-phosphate of zinc to prevent pulpitis, which might be occasioned by the expansion and consequent pressure of gutta-percha stoppings. Similar care should also be observed where the enamel-walls are so exceedingly frail as to become easily fractured.

Although these stoppings are liable to wear away when much exposed to attrition, the surrounding cavity-walls usually remain well preserved. They are, moreover, easily repaired or renewed, and with no loss to the tooth structure.

For large stoppings, much exposed to wear, caps of gold plate can be fitted to cover them accurately, on the cavity surface of which may be soldered small loops or "T"-shaped anchors. Such a cap, warmed over a spirit-lamp, can be imbedded in, or united with the fillings, leaving a firm gold surface on which to masticate.

With a degree of tact and experience, gutta-percha stoppings can be manipulated readily and with comparatively little trouble. Cavities should be prepared as nicely as possible, and kept dry while filling is introduced.

Small pellets of the stopping heated to a plastic condition, can be carried to the cavity on the point of a small curved and flattened instrument. Gentle pressure against the walls packs it securely. The excess can be trimmed away with flat heated instruments, and the surface rubbed with burnishers. A bit of cotton or spunk moistened with chloroform, held with tweezers and passed over the filling, will also aid in smoothing it.

Great care is requisite to avoid over-heating the material. If warmed over a spirit-lamp it must be held considerably above the flame. It is safer to place bits of the stopping on a piece of heated porcelain, or a small covered vessel of boiling water, preparatory to use.

Gutta-percha stoppings, if well impacted in properly prepared cavities, seldom prove treacherous, but as a rule are exceedingly safe and reliable.

